# Rapid Typing of Transmissible Spongiform Encephalopathy Strains with Differential ELISA

**DOI:** 10.3201/eid1404.071134

**Published:** 2008-04

**Authors:** Stéphanie Simon, Jérôme Nugier, Nathalie Morel, Hervé Boutal, Christophe Créminon, Sylvie L. Benestad, Olivier Andréoletti, Frédéric Lantier, Jean-Marc Bilheude, Muriel Feyssaguet, Anne-Gaëlle Biacabe, Thierry Baron, Jacques Grassi

**Affiliations:** * Commissariat à l’Energie Atomique, Gif-sur-Yvette, France; † National Veterinary Institute, Oslo, Norway; ‡ Institut National de la Recherche Agronomique-Ecole Nationale Vétérinaire de Toulouse, Toulouse, France; § Infectiologie Animale et Santé Publique, Tours, France; ¶ Bio-Rad, Marnes-la-Coquette, France; # Agence Française de Sécurité Sanitaire des Aliments, Lyon, France

**Keywords:** PrP, BSE, scrapie, ELISA, strain typing, research

## Abstract

The bovine spongiform encephalopathy (BSE) agent has been transmitted to humans, leading to variant Creutzfeldt-Jakob disease. Sheep and goats can be experimentally infected by BSE and have been potentially exposed to natural BSE; however, whether BSE can be transmitted to small ruminants is not known. Based on the particular biochemical properties of the abnormal prion protein (PrPsc) associated with BSE, and particularly the increased degradation induced by proteinase K in the N terminal part of PrPsc, we have developed a rapid ELISA designed to distinguish BSE from other scrapie strains. This assay clearly discriminates experimental ovine BSE from other scrapie strains and was used to screen 260 transmissible spongiform encephalopathy (TSE)–infected small ruminant samples identified by the French active surveillance network (2002/2003). In this context, this test has helped to identify the first case of natural BSE in a goat and can be used to classify TSE isolates based on the proteinase K sensitivity of PrPsc.

## Abstract 2

A strain-typing ELISA distinguishes bovine spongiform encephalopathy from other scrapie strains in small ruminants.

Transmissible spongiform encephalopathies (TSEs) are neurodegenerative diseases affecting humans and animals; examples are scrapie in sheep and goats, bovine spongiform encephalopathy (BSE) in cattle, and chronic wasting disease in cervids. Strong evidence indicates that the BSE agent has been transmitted to humans through the food chain, leading to the emergence of variant Creutzfeldt-Jakob disease (vCJD). Recently, the first case of natural BSE was identified in a French goat (*1*), and a second probable one was reported in the United Kingdom (*2*). It seems, however, that these were isolated cases and that the BSE level in small ruminant flocks is probably low. However, BSE can be transmitted experimentally to sheep and goats by the oral route (*3*–*5*), whereas small ruminants have very likely been exposed to the BSE agent through contaminated meat and bone meal (MBM). Given the widespread tissue distribution of the BSE agent in sheep and goats, horizontal and vertical transmission within sheep and goat populations is possible, which could lead to human exposure to the agent through the food chain, even after the MBM feed ban.

TSEs are characterized by the accumulation in the brain of an abnormal form of the prion protein (PrPsc), which is derived from the normal one (PrPc) and manifests as unusual biochemical properties including insolubility in the presence of detergents and partial resistance to the action of proteases like proteinase K (PK). The conventional method of identifying a TSE strain is the experimental transmission of the disease in several mouse lines (*6*,*7*) including transgenic mouse lines that over express PrP, thereby increasing the efficiency and speed of transmission (*8*–*10*). However, these methods cannot be used as screening methods.

Sophisticated histopathologic and immunohistochemical methods have also been used to identify TSE strains (*5*,*11*–*15*). These methods appear efficient in differentiating BSE from other scrapie strains, but they are time-consuming and not suitable for large-scale typing.

Several studies have shown that the resistance of PrPsc to PK treatment varies depending on the strain (*16*–*18*). PrPsc associated with BSE in cattle or sheep is more sensitive to PK degradation at its amino-terminus than PrPsc from scrapie. Recently “atypical” forms of scrapie sensitive to PK, such as the Nor98 strain, have been described (*19*). Since 2002, large-scale epidemiologic studies have led to the identification of hundreds of atypical cases in several European countries (*20*); these cases included an unusual occurrence in sheep with genotypes previously associated with high resistance to scrapie (including the ARR/ARR genotype) (*21*). Converging data show that most atypical cases share identical biochemical and biologic features with Nor-98 (*22*).

Glycoform ratios and the molecular migration pattern of PK-treated PrPsc, as obtained in Western blot techniques, enable characterization of scrapie strains and BSE. (These topics have been reviewed by Groschup et al. [*23*]) This method identified similarities between BSE in cattle and experimental BSE in sheep (*24*,*25*) and similarities between vCJD and BSE in cattle (*26*,*27*). However, these typing immunoblotting techniques are not easily applied to test huge numbers of field samples. To increase specificity, a differential immunoblotting technique involving 2 specific antibodies, directed against the amino- and carboxy-termini of PrP, has been developed (*10*,*28*). Comparison of the signals obtained for both antibodies provides an easy and fast identification of the BSE strain because binding of the anti-N terminal antibody is almost completely suppressed. This approach has been used to characterize unusual BSE cases in cattle (*29*) and naturally infected scrapie sheep (*14*,*30*).

We describe an ELISA designed to distinguish BSE and scrapie strains, on the basis of their differential resistance to PK. The design of this method is similar to that of a rapid test widely used in Europe (TeSeE, Bio-Rad, Hercules, CA, USA) and allows rapid screening of a large number of sheep samples previously shown to contain PK-resistant prion protein (PrPres) by a conventional test.

## Experimental Procedures

### Materials

Chemicals were obtained from Sigma (St. Louis, MO, USA) except (aminoethyl)benzenesulfonyl fluoride) (AEBSF) from ICN Pharmaceuticals, Inc. (Costa Mes, CA, USA). All other reagents for purification and detection of PrPres were from the Bio-Rad TeSeE and TeSeE Sheep & Goat kits.

### Samples

#### Experimental Samples

Experimental samples for scrapie controls 99–1316 and 99–1487 were from the Veterinary School of Toulouse (France) and for PG1259 from the Veterinary Laboratories Agency (VLA, Weybridge UK). Experimental ovine BSE-infected samples (SB1 and SB3 [*31*]) were from Agence Française de Sécurité Sanitaire des Aliments (AFSSA), the French national reference laboratory in Lyon. Some of the first-passage experimental ovine BSE-infected animals (at the clinical stage of the disease) were produced by UR INRA 1282 Nouzilly (intracerebral inoculation) in the framework of the European “BSE in sheep” QLKCT 2001–01309 program (nos. 347, 359, 378, 384, 386, 388, 397, bearing ARQ/ARQ genotype; nos. 12, 38, 331, 337, 341, 368, and 369 had the ARR/ARR genotype). First-passage experimental ovine BSE-infected animals 7704, 7705, PG0637/01, PG0638/01, PG0639/01, and PG0640/01 as well as second-passage animals 1630M, 1631M, 1636M, 1637M, 1638M, 1639M, 1641M, 1643M, 1644M, and 1645M were from VLA. Experimental caprine BSE samples were from the Institute for Animal Health, Edinburgh, Scotland. Nor98 isolates were from the National Veterinary Institute, Oslo, Norway.

#### Field Isolates

This study tested, in blind conditions, 270 samples provided by AFSSA. This represents all the TSE-positive cases recorded during 2002–2003 by active surveillance (>160,000 brain samples originating from slaughterhouse and rendering plants screened by rapid tests) and is thus representative of the diversity of French isolates. The 270 samples included 21 caprine isolates and 42 cases of atypical scrapie previously identified by AFSSA.

### Homogenization of Nervous Tissues

Nervous tissues were homogenized and calibrated according to the Bio-Rad purification protocol. Homogenates (20% w/v) were diluted 10-fold in a negative sheep brain homogenate or tested undiluted if absorbance measurement in A reagent was <0.5.

### Processing of Samples (Scrapie-associated Fibrils Preparation)

Two batches (A and A′series) of 21 samples (250 μL (20% homogenate) of each sample per tube, manual procedure) or 29 samples (250 μL of each sample per well in 2 deep-well plates, automated protocol) were analyzed together with 2 scrapie controls (nerve tissue from 2 scrapie sheep, 99–1316 and PG1259 [manual] or 99–1316 and 99–1487 [automated]) and an experimental BSE-infected sheep (397BS, INRA). Each batch was treated in 1 set of conditions of PK treatment. For the manual protocol, 250 μL of A reagent (TeSeE purification kit) containing PK (10 μg) is distributed in each tube of the A series, and 250 μL of A′reagent/PK (5% N-lauroylsarcosine sodium salt [w/v], 5% [w/v] sodium dodecyl sulfate [SDS] containing 27.5 μg of PK) in each tube of the A′series. All the tubes were then homogenized by 10 inversions and incubated at 37°C for 15 min. Then, 250 μL of B reagent (Bio-Rad purification kit)/phenylmethylsulfonyl fluoride (PMSF) (final concentration 4 mmol/L) was added, before homogenization and centrifugation for 5 min at 20,000× *g* at 20°C.

For the automated protocol, the deep-well plates were successively incubated for 12 min at 37°C in the TeSeE NSP automated system. Each well of the first plate was processed with 250 μL of A reagent/PK before 15 min of incubation at 37°C. Then 250 μL of the B reagent containing PMSF (12 mmol/L) was added and incubated 5 min at 37°C. The deep-well plate was centrifuged for 10 min at 2,000 × *g*, 4°C. The second plate (A′series) was processed similarly with the A′reagent/PK.

For both protocols, supernatants were discarded and the tubes (or the plates) dried by inversion on absorbent paper for 5 min. Each pellet was denatured for 5 min at 100°C with 25 μL of C reagent (Bio-Rad purification kit). We then added 350 μL of R6 buffer containing 4 mmol/L AEBSF. For the field isolates, serial dilutions (3- and 10-fold in R6 buffer/AEBSF) were performed to ensure an optimal ELISA signal.

### Immunometric Assay

All the reagents were provided by the Bio-Rad TeSeE Sheep & Goat detection kit. We distributed, in duplicate, 100 μL of samples (undiluted, 3- and 10-fold diluted) and controls in microtiter plates coated with the first anti-PrP antibody. The plate reacted for 2 h at room temperature (RT). After 3 washing cycles (R2 buffer), 100 μL/well of the enzyme conjugate was added for 2-h reaction at RT. After 3 washing cycles, 100 μL of substrate was added for 30 min in the dark at RT, before blocking the reaction with 100 μL of stopping solution and reading the absorbance at 450/620 nm. The mean ratio of the absorbances obtained in the 2 conditions (A and A′) was calculated for each sample by selecting a range of appropriate dilutions providing absorbance measurements ranging from 0.5 to 2.5 for A reagent.

### Immunoblot

Pellets of purified PrPres (TeSeE purification kit) were denatured in Laemmli buffer for 5 min at 100°C. After SDS–12% PAGE, samples were blotted on polyvinylidene difluoride membranes (Bio-Rad) and blocked with 5% bovine serum albumin. PrP was detected by using horseradish peroxidase–labeled SHa31 antibody and chemiluminescence (ECL plus Western blotting detection system, Amersham Biosciences, Piscataway, NJ, USA).

### PK Range for Nor98 Isolates

Five Nor98 isolates as well as the 2 scrapie controls and the experimental ovine BSE were diluted in ovine negative brain homogenate (200- to 800-fold). In 6 sets of PK conditions 250 μL of each sample was treated: 1 with 250 μL of A TeSeE reagent (TeSeE purification kit) with 10 μg of PK and 5 different PK concentrations (10 μg, 15 μg, 20 μg, 25 μg, and 30 μg) in 250 μL of A TeSeE Sheep & Goat reagent, named A′′ reagent (TeSeE Sheep & Goat purification kit).

## Results

### Principle of the Typing Test

The typing ELISA is based on the screening test for the postmortem diagnosis of BSE initially developed by the Commissariat à l’Energie Atomique (CEA) (*32*,*33*). After selective purification of PrPres, the denatured PrPres is measured by using a sandwich assay in microtiter plates.

**Figure 1:**
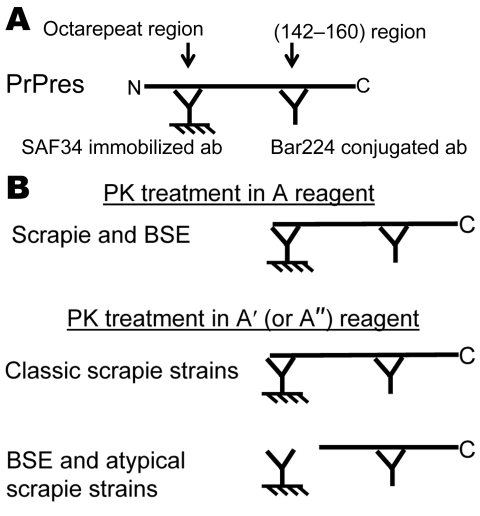
Principle of the 2-site immunometric typing assay. A) EIA screening test. In these conditions (Bio-Rad, Hercules, CA, USA), after mild proteinase K (PK) digestion, denatured PK-resistant prion protein (PrPres) is captured by the solid phase–immobilized antibody SAF34, recognizing the octarepeat region, and shown by the Bar224 tracer antibody, directed against the core of the protein. B) EIA typing test. In this test, the PrPres-containing sample is treated in 2 sets of PK conditions. In the first set of conditions (PK treatment in A reagent, Bio-Rad screening condition), the octarepeat region is maintained in scrapie- and bovine spongiform encephalopathy (BSE)–associated PrPres; in the second set of conditions (high PK concentration in A′reagent), BSE- and labile strain–associated PrPres is more sensitive to PK digestion than the classic PrPres associated with scrapie strains. Calculation of the ratio in the 2 conditions differentiates BSE and labile strains from classic scrapie strains. ab, antibody.

The capture antibody recognizes an amino terminal epitope, while the tracer antibody binds to the core of the protein (Figure 1, panel** A**). In the first set of conditions, the PK digestion is performed in a control medium (mixture of detergents and chaotropic agents) to preserve the N-terminal epitope (Figure 1, panels** A** and **B**, PK treatment in A conditions). By varying the conditions of PK treatment, PrPsc associated with the BSE strain appears more sensitive to PK than most of the other prion strains. Conditions of PK treatment can thus be defined for selectively deleting the epitope recognized by the capture antibody in BSE strain while preserving it in most scrapie strains (Figure 1, panel** B**, PK in A′conditions), in agreement with previous reports (*5*,*10*,*23*,*28*).

**Figure 2:**
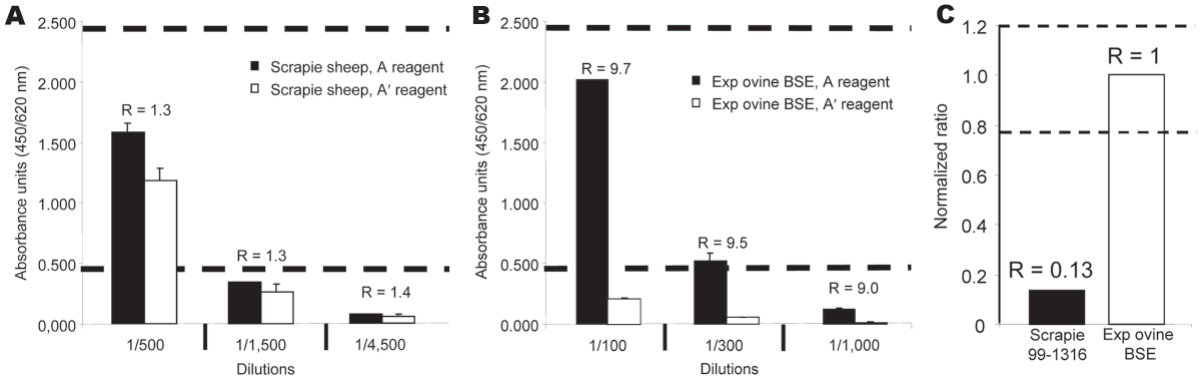
Determination of the A/A’ ratio. A dilution series was assayed for each analyzed sample to determine the optimal range that would permit precise determination of the A/A′ratio (absorbance ranging from 0.5 to 2.5 absorbance units in A reagent). A) A/A′ratio is close to 1 for PK-resistant prion protein (PrPres) associated with classic scrapie strains (manual protocol, see Experimental Procedures) and B) close to 10 for experimental ovine bovine spongiform encephalopathy (BSE)–associated PrPres. C) To minimize interassay variations, the ratio obtained for each sample is thus normalized by dividing by the ratio obtained for the ovine BSE sample.

The first step of the Bio-Rad test (purification and concentration of PrPres) was performed either manually or by using the NSP automated system and adapted for the typing test. Determination of the ratio between the 2 measurements (R = A/A′) allows differential detection of the BSE strain. In the current conditions, this ratio is <2 for most of the scrapie strains (manual or automated protocol) and close to 6 and 10 for the automated and manual protocols, respectively, with the BSE strain (Table 1). During the current study, we made 2 major changes with regards to the Bio-Rad test: 1) we added PK inhibitors (PMSF and AEBSF) at 2 stages of the purification procedure for better control of PK digestion, and 2) we performed a series of dilutions to determine the optimal range of absorbance, which allowed a precise determination of the A/A′ratio (ranging between 0.5 and 2.5 absorbance unit, Figure 2, **panels A** and **B**). To minimize the interassay variations, the ratio obtained for each sample was further normalized by dividing it by the ratio obtained for the experimental ovine BSE control (Figure 2, panel** C**)

**Table 1:** Analysis of the reproducibility of the ELISA typing test*

Sample (N = 20)	Mean ratio A/A′(normalized)	SD (normalized)	CV, %	95% CI (mean ± 2× SD)
Manual protocol				
Classic scrapie 99-1316	1.3 (0.13)	0.2 (0.02)	15.4	0.9–1.7 (0.09–0.18)
Intermediate scrapie PG1259	4.5 (0.46)	0.8 (0.08)	17.8	2.9–6.1 (0.30–0.63)
Experimental ovine BSE 397BS	9.7 (1.00)	2.0 (0.21)	20.6	5.7–13.7 (0.59–1.41)
Automated protocol				
Classic scrapie 99-1316	1.6 (0.25)	0.2 (0.03)	13.7	1.2–1.9 (0.19–0.30)
Intermediate scrapie 99-1487	3.8 (0.59)	0.5 (0.08)	13.4	2.8–4.8 (0.44–0.75)
Experimental ovine BSE 397BS	6.4 (1.00)	0.9 (0.14)	14.8	4.5–8.3 (0.70–1.30)

*Three samples (classic scrapie 99-1316, intermediate scrapie PG1259 or 99-1487, and experimental ovine BSE 397BS) were analyzed in 20 different experiments using the manual or automated protocol (see Experimental Procedures). Normalized ratios are indicated in parentheses. SD, standard deviation; CV, coefficient of variation; CI, confidence interval; BSE, bovine spongiform encephalopathy.

To evaluate this typing test, 37 brain samples from 25 ARQ/ARQ sheep experimentally infected with BSE (first or second passage) were compared with the brain samples of 3 controls. All samples analyzed in 3 independent experiments provide A/A′ratios >7 with a mean of 11 0.2 ± 1.4 (Appendix Figure 1, panel** A**). To further investigate the possible influence of the genotype, we tested 7 spinal cord samples from experimental BSE bearing the ARR/ARR genotype, in at least 4 independent experiments. A/A′ratios ranged from 5.5 to 6.9 (mean 6.0 ± 0.4) and were statistically different from the experimental ARQ/ARQ ovine BSE used as control (mean 8.8 ± 0.9, p<0.001 for all samples except no. 38, p<0.05) (Appendix Figure 1, panel** B**).

### Specificity of Typing Test

We analyzed a large series of field isolates identified as TSE infected, by a rapid test, in the framework of the French active surveillance network. Of these 270 samples (153 in 2002 and 117 in 2003), 42 samples, which were initially identified by using the TeSeE test, were categorized by AFSSA as atypical due to lack of confirmation by other rapid tests or World Organization for Animal Health–modified SAF immunoblot (AFSSA, no. 2004-SA-0045) (*34*).

**Figure 3:**
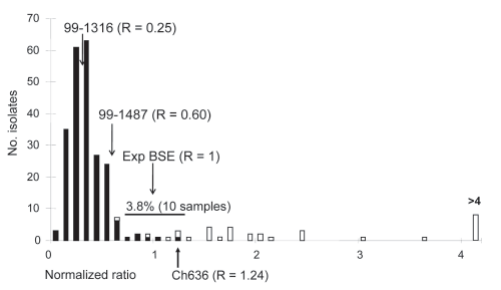
A total of 260 French field-positive isolates were analyzed with the automated ELISA typing test. Each series of 29 samples was analyzed together with 3 internal controls (classic scrapie, intermediate scrapie, experimental ovine bovine spongiform encephalopathy [BSE]). The ratio obtained for each sample was normalized by dividing by the experimental ovine BSE in the same series. Open bars represent the atypical scrapie cases. Natural goat BSE isolate (Ch636) is indicated.

When tested with the automated typing test, 10 of the 270 samples had PrPres levels below the detection limit, even undiluted (optical density <0.5 in A conditions). Each series of 32 samples included 3 internal controls (see Table 1 for results obtained for 20 different series of tests). Among the 2 scrapie controls, 1 (99–1316) was classified as classic as it has a PK resistance similar to most of the field isolates; the second one (PG1259 or 99–1487) was classified as intermediate because its PK resistance was intermediate between classic scrapie and experimental ovine BSE. The distribution of the normalized ratio recorded with the field isolates is shown in Figure 3. The absorbance ratio under the 2 conditions of treatment was remarkably reproducible for the classic scrapie control (mean 1.6 ± 0.2), while larger variations were observed for experimental ovine BSE and the intermediate scrapie control (mean 6.4 ± 0.9 and 3.8 ± 0.5, respectively). These results demonstrate that the present test clearly discriminates classic scrapie strain from the 2 other controls. Because of interassay variations, experimental ovine BSE and intermediate scrapie may slightly overlap (Table 1), further justifying the use of normalized ratio (Figure 2, panel** C**, and Figure 3).

**Figure 4:**
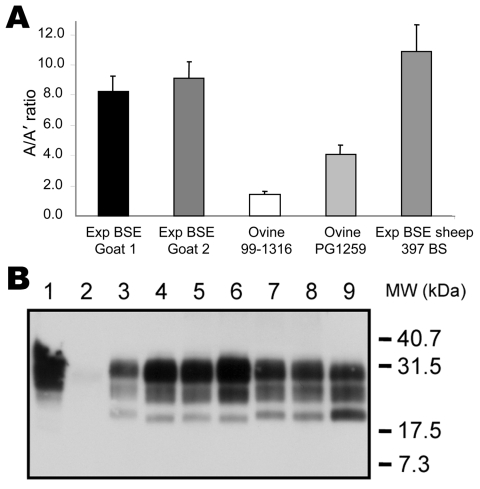
Analysis of goats isolates with ELISA typing test and immunoblot. Two experimental bovine spongiform encephalopathy (BSE) samples in goats analyzed by using the manual typing ELISA (A) gave ratios similar to those of experimental BSE in sheep. Results are the mean of 3 independent experiments. Experimental goat BSE and BSE-like field isolate Ch636 were analyzed by Western blot and compared with scrapie goat isolates (B). Lane 1, untreated negative brain homogenate. Lane 2, proteinase K (PK)–treated negative brain homogenate. Lanes 3–9, PK-treated positive isolates: French scrapie isolate 99–1316 (lane 3); experimental ovine BSE 397 BS (lane 4); experimental BSE goat 2 (lane 5); French goat isolate Ch 636 (campaign 2002) (lane 6); French scrapie goat isolates Ch517 and Ch519 (campaign 2002) (lanes 7 and 8, respectively); Norwegian scrapie isolate (Lavik, lane 9). MW, molecular weight.

As shown in Figure 3, only 10 samples (3.8%) provided ratios compatible with experimental BSE (normalized ratio 0.7–1.3), and 28 provided ratios superior to experimental BSE in sheep (normalized ratio >1.3–10.5, Figure 3). Twenty-nine samples, as well as 4 of the 10 samples compatible with experimental BSE and 9 samples with low levels of PrPres, were previously identified as atypical scrapie (open bars, Figure 3). Among the samples that yielded ratios compatible with experimental BSE in sheep, 1 goat isolate, Ch636, was extensively studied because of its BSE-like profile in 3 different Western blot techniques (*1*). As shown in Figure 3, this sample had a normalized A/A′ratio close to 1, very similar to that for experimental goat BSE (Figure 4, panel** A**, Table 2). This result is confirmed by the migration pattern of the nonglycosylated band (Figure 4, panel **B**, lane 6), which appears very similar to that of experimental BSE in sheep (Figure 4, panel** B**, lane 4) and of experimental goat BSE (lane 5), and different from that of French scrapie goat isolates (lanes 7 and 8).

**Table 2:** Analysis of the experimental caprine BSE samples using the manual ELISA typing test*

Samples	Mean A/A′ratio (normalized)	SD (normalized)	CV, %	95% CI (mean ± 2×SD)
Experimental BSE goat 1	8.2 (0.75)	1.0 (0.09)	12.2	6.2–10.2 (0.57–0.93)
Experimental BSE goat 2	9.1 (0.83)	1.1 (0.10)	12.1	6.9–11.3 (0.63–1.04)
99-1316	1.5 (0.14)	0.2 (0.02)	13.3	1.1–1.9 (0.10–0.17)
PG1259	4.1 (0.38)	0.6 (0.06)	14.6	2.9–5.3 (0.27–0.49)
Experimental ovine BSE 397BS	10.9 (1.00)	1.8 (0.17)	12.2	7.3–14.5 (0.67–1.33)

*BSE, bovine spongiform encephalopathy; SD, standard deviation; CV, coefficient of variation; CI, confidence interval.

### Analysis of Nor98 Isolates

**Figure 5:**
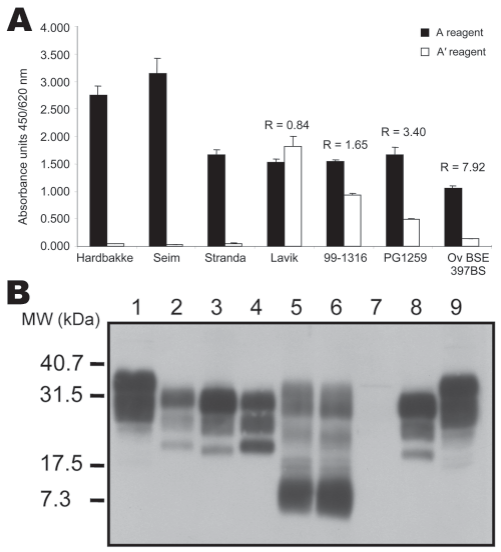
Analysis of different ovine strains by ELISA typing test and immunoblot. A) ELISA typing test. Three Nor98 isolates (Fiksdal, Stranda, and Seim) were analyzed by using the ELISA typing test. Absorbances obtained in the classic A′typing reagent are close to 0, preventing calculation of the A/A′ratio. Ratios obtained for a Norwegian scrapie isolate (Lavik) and for the 3 internal controls (classic Scr 99–1316, intermediate scrapie PG1259, and experimental ovine bovine spongiform encephalopathy [BSE] 397 BS) are indicated. B) Pattern of migration of different ovine strains. Lanes 1 and 9, untreated negative brain homogenate. Lanes 2–8, proteinase K–treated brain homogenates: French scrapie isolate 99–1316 (lane 2); experimental ovine BSE 397 BS (lanes 3 and 8); Norwegian scrapie isolate (Lavik) (lane 4); Nor98 Stranda and Nor98 Seim scrapie isolates (lanes 5 and 6, respectively); negative brain homogenate (lane 7). MW, molecular weight.

The typing test was used to analyze 18 sheep isolates from Norway (Table 3). Ratios were almost impossible to calculate because of the large decrease in signal in A′conditions, as shown in Figure 5, panel** A** for 3 isolates. Only 1 sample (Lavik) showed characteristics of a conventional scrapie isolate, providing an A/A′ratio of 0.84 (Figure 5, panel** A**), a normalized ratio of 0.11, and a Western blot profile close to that of a French scrapie isolate (Figure 4, panel** B**, lanes 3 and 9; Figure 5, panel** B**, lanes 2 and 4). Other samples had a pattern that included a 12-kDa band (Figure 5, panel** B**) (*19*,*22*,*34*), characteristic of the Nor-98 strain.

**Table 3:** Normalized ratios obtained for the Norwegian isolates and controls*

No.	Name	Normalized ratio	SD
Isolates			
Nor 98 1	Andoya	2.51	0.58
Nor 98 4	Fiksdal	4.22	1.34
Nor 98 5	Gasbakken	2.73	0.50
Nor 98 6	Hardbakke	3.81	0.76
Scr 7	Lavik	0.42	0.14
Nor 98 8	Lindas	1.43	0.40
Nor 98 9	Lom	5.30	1.37
Nor 98 10	Narvik	10.43	3.56
Nor 98 11	Oppdal	1.79	0.24
Nor 98 12	Rauland	4.28	1.00
Nor 98 13	Rennebu	4.23	0.55
Nor 98 14	Seim	3.16	0.63
Nor 98 16	Stranda	1.90	0.26
Nor 98 17	Torsvastad	3.45	0.33
Nor 98 19	Al	5.31	3.27
Nor 98 20	Arnes	3.99	0.85
Nor 98 21	Aseral	3.20	0.67
Nor 98 22	Egersund	1.68	0.52
Nor 98 24	Soknedal2	14.22	6.21
Nor 98 26	Tennevoll	95.43	90.01
Nor 98 27	Sortland	6.27	1.68
Controls			
Ov 99-1316		0.36	0.11
PG-1259		0.45	0.14
BSE 397BS		1.00	0

*SD, standard deviation; BSE, bovine spongiform encephalopathy.

**Figure 6:**
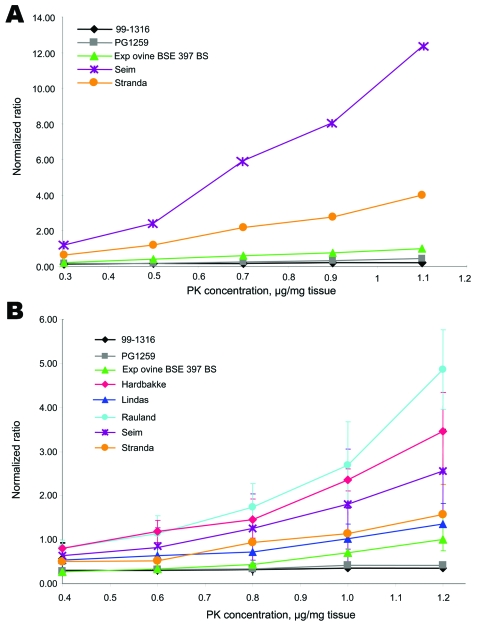
Proteinase K (PK) sensitivity of Nor98 isolates in stringent and mild detergent conditions. The ELISA typing test was performed on Nor98 isolates, with 5 concentrations (0.4–1.1 µg per mg of tissue) in the stringent A′reagent (A) or in the mild A′′ reagent, adapted for PK-sensitive strains (B) (see Experimental Procedures). A/A′(or A/A′′) ratios were calculated for each PK concentration, and normalized by dividing by the A/A′ratio (or A/A′′) obtained for the experimental ovine bovine spongiform encephalopathy (BSE) sample at the maximal PK concentration. In the A’ reagent, even at the lowest PK concentration (PK 0.4 μg/mg tissue), the normalized ratios (using the experimental ovine BSE A/A′PK1.1 ratio) obtained for the Nor98 isolates are >1, thus being 3× more sensitive than experimental ovine BSE. To evaluate possible differences in PK sensitivity among Nor98 isolates, this experiment was reproduced with the A′′ reagent (panel B), which is 3- to 6-fold more protective than the A′reagent, as shown by the corresponding normalized ratios (A′or A′′ reagent) for the same PK concentration (1.1 μg PK/mg of tissue).

After adapting the conditions of the PK treatment in the second set of measurements (A′conditions), we observed (see legend, Figure 6) a much lower A/A′ratio for those Nor-98, which enables discrimination of highly sensitive PK samples (nos. 24 and 26, Appendix Figure 2 and Table 3) to mildly sensitive PK samples (nos. 8, 11, 16, and 22).

## Discussion

When this study was initiated, no case of natural BSE in small ruminants was recorded, and only a few experimental ovine BSE samples were available, all belonging to the same PrP genotype (ARQ/ARQ), and mainly from a first passage. The possible impact of the genotype, the route of infection, and the number of passages on the biochemical properties of PrPres associated with the BSE strain are poorly understood. Now, further data suggest that, at least during the second passage in sheep, the biochemical properties (glycoform pattern in brain) of the BSE agent are unchanged (*35*,*36*). In this study using our ELISA, small ruminant BSE samples clearly behaved differently from conventional scrapie samples. However, slight differences may exist (see ARQ/ARQ vs. ARR/ARR genotype in Appendix Figure 1). We do not know whether these findings reflect differences in the PK sensitivity of PrPres associated with these genotypes or the influence of different tissues.

The main difficulty encountered for the development of a typing test is evaluation of its specificity and sensitivity. In the current study, we unambiguously identified all 37 experimental ovine BSE samples from 25 sheep, including 10 from a second passage. There are few data describing the molecular features of PrPres associated with experimental BSE in goats (*37*,*38*). In the framework of the French scrapie strain-typing network, 18 goats were analyzed by this ELISA, and 2 appeared compatible with experimental ovine and caprine BSE. One of them (Ch636), when analyzed with other molecular typing tests, appeared indistinguishable from experimental BSE and was later confirmed as the first natural case of BSE in a goat (*1*), after experimental transmission in wild-type and transgenic mice. The second BSE compatible sample (TR041528) was later clearly identified as a case of atypical scrapie as defined by its migration pattern (*34*). All these data suggest a good sensitivity for our test, which unambiguously identified all cases of experimental BSE in the sheep and goats tested, as well as the only natural case identified to date in a goat.

Another key point during the development of this test was to ensure good reproducibility because this parameter clearly influences both sensitivity and specificity. Ratios obtained for the classic scrapie control were highly reproducible, whereas ratios measured for the experimental BSE in sheep and the intermediate scrapie control varied much more, leading to an overlap of the 95% confidence interval (Table 1). To minimize interassay variations, the ratio obtained for each unknown sample was thus normalized by taking as reference the ratio measured for the ovine BSE sample (Figure 2, panel** C**, and Figure 3) in the same experiment. This enabled us to define the range of normalized ratio compatible with BSE as the mean of experimental ovine BSE ± 2σ on the basis of reproducibility experiments recorded in Table 1. This range was experimentally determined between 0.7 and 1.3, leading to 3 categories for field samples: conventional scrapie (ratio <0.7), compatible with BSE (0.7 <ratio <1.3), and atypical scrapie (ratio >1.3).

Only 10 (3.8%) of the 260 samples analyzed in the framework of the French epidemiologic surveillance network during 2002–2003 gave a ratio compatible with BSE. Of the 10 BSE suspected samples, only 1 goat sample (Ch636) was later confirmed as a true natural BSE case (*1*). This result indicates that the specificity of this test is not that good because 9 false-positive results were recorded in 260 samples (specificity 96.5%). However, the test appears useful since it excluded the presence of BSE for most field samples, thus restricting the use of more specific but time-consuming methods, like experimental transmission in mice, to a small number of isolates. Moreover, in a single screening, this test classified all TSE-infected isolates as a function of their PK resistance and thus provided a rapid classification of sheep isolates according to this criterion. The test could also be modified, by adjusting the range of PK sensitivity, to classify Nor-98 isolates.

All these data demonstrate that this ELISA-based typing test is suitable for a routine analysis of field samples, as assessed by the positive evaluation from the European Commission as one of the tests recommended to identify the possible presence of BSE in small ruminant flocks (http://eur-lex.europa.eu/LexUriServ/site/en/oj/2005/l_010/l_01020050113en00090017.pdf). These typing tests are mainly designed to identify the BSE strain in small ruminant flocks. They are performed exclusively in national reference laboratories and based on Western blot techniques. In this context, the present ELISA is one of the secondary tests to be used to confirm BSE suspicion. We believe it will help clarify the status of these unusual isolates.

## Supplementary Material

Appendix Figure 1 Sensitivity of the ELISA typing test. A) ARQ/ARQ genotype. Fifteen experimental ovine bovine spongiform encephalopathy (BSE) animals from a first passage and 10 from a second passage were tested by using the typing ELISA, in at least 3 independent experiments. For some animals, different regions of the central nervous system were tested (animals SB1, 359, 397, 7704, and 7705). SC, spinal cord. BS, brain stem; FC, frontal cortex; PC, parietal cortex; OC, occipital cortex. B) ARR/ARR genotype. Seven experimental ovine BSE animals from a first passage were tested by using the typing ELISA, in at least 4 independent experiments.

Appendix Figure 2 Proteinase K (PK) sensitivity of 21 Norwegian isolates in mild conditions. The ELISA typing test was performed on 20 Nor98 isolates and 1 Norwegian scrapie isolate (Lavik) by using the protective A´´ reagent, with a PK concentration of 1.2 µg/mg tissue. These new conditions (PK 1.2 µg/mg of tissue in A´´ reagent) showed a large range of PK resistance with normalized ratios varying from 1.4 for the most resistant (Lindas isolate), close to the experimental ovine BSE, to >14 for the weakest (Soknedal2). One isolate, Tennevoll, appeared so sensitive to PK digestion that the ratio could not be evaluated (>95) even using these protective conditions.
