# Supplementary figures and images for: Rapid Typing of Transmissible Spongiform Encephalopathy Strains with Differential ELISA
Source: Emerg Infect Dis. 2008 Apr;14(4):608–16. doi: 10.3201/eid1404.071134 (PMC2570920; doi:10.3201/eid1404.071134)

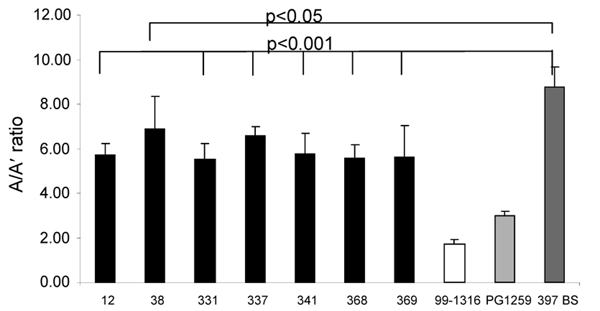

Supplement: Appendix Figure 1 — Sensitivity of the ELISA typing test. A) ARQ/ARQ genotype. Fifteen experimental ovine bovine spongiform encephalopathy (BSE) animals from a first passage and 10 from a second passage were tested by using the typing ELISA, in at least 3 independent experiments. For some animals, different regions of the central nervous system were tested (animals SB1, 359, 397, 7704, and 7705). SC, spinal cord. BS, brain stem; FC, frontal cortex; PC, parietal cortex; OC, occipital cortex. B) ARR/ARR genotype. Seven experimental ovine BSE animals from a first passage were tested by using the typing ELISA, in at least 4 independent experiments. [file 07-1134_app1.gif]

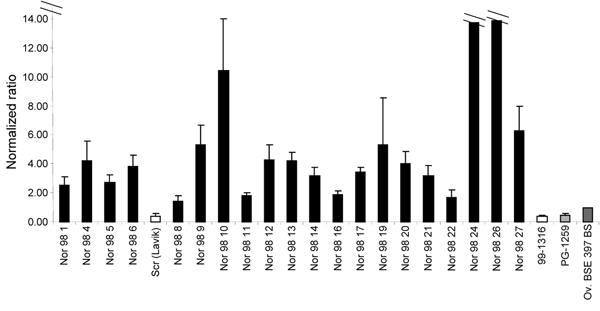

Supplement: Appendix Figure 2 — Proteinase K (PK) sensitivity of 21 Norwegian isolates in mild conditions. The ELISA typing test was performed on 20 Nor98 isolates and 1 Norwegian scrapie isolate (Lavik) by using the protective A´´ reagent, with a PK concentration of 1.2 µg/mg tissue. These new conditions (PK 1.2 µg/mg of tissue in A´´ reagent) showed a large range of PK resistance with normalized ratios varying from 1.4 for the most resistant (Lindas isolate), close to the experimental ovine BSE, to >14 for the weakest (Soknedal2). One isolate, Tennevoll, appeared so sensitive to PK digestion that the ratio could not be evaluated (>95) even using these protective conditions. [file 07-1134_app2.gif]
